# Primary Ciliary Dyskinesia: A Suggested Pathway to Transition From Pediatric to Adult Care

**DOI:** 10.1002/ppul.71780

**Published:** 2026-08-13

**Authors:** Abel De Castro, Melanie Sue Collins, Raksha Jain, Jennifer B. Walsh, Juliana Alba‐Suárez, Yadira M. Rivera‐Sánchez

**Affiliations:** ^1^ University of Texas Southwestern Medical Center Dallas Texas USA; ^2^ University of Connecticut School of Medicine/Connecticut Children's Hartford Connecticut USA; ^3^ University of Texas Southwestern Medical Center/Dallas Children's Health Dallas Texas USA

## Abstract

Primary ciliary dyskinesia (PCD) is a rare, genetically heterogeneous disorder characterized by motile ciliary dysfunction that impairs mucociliary clearance. Symptoms of PCD can include unexplained neonatal respiratory issues, chronic sino‐otopulmonary symptoms, recurrent infections, bronchiectasis, and subfertility. It is expected that the number of adult patients with PCD is four times higher than the number of pediatric patients. There are no established guidelines for transitioning individuals with PCD from pediatric to adult clinics. Although the exact prevalence is difficult to determine, it is estimated that up to 13% of adults with non‐CF bronchiectasis have PCD. The Six Core Elements of Health Care Transition, developed by Got Transition, funded by the National Alliance to Advance Adolescent Health, and endorsed by the American Academy of Pediatrics, the American Academy of Family Physicians, and the American College of Physicians, can serve as a framework for transitioning PCD patients to adult care. Programs implementing the Six Core Elements have significantly reduced care dropouts during adolescence and early adulthood and increased engagement in care in early adulthood. PCD has been the subject of growing research aimed at improving diagnostic methods, treatments, and awareness, leading to earlier and more accurate diagnoses in both children and adults. Additionally, more adult PCD centers are seeking accreditation from the PCD Foundation and collaborating with pediatric centers to ensure continuity of care. Although many chronic diseases, such as cystic fibrosis (CF), inflammatory bowel disease (IBD), and, more recently, sickle cell disease, have established transition guidelines, PCD presents unique challenges that affect the transition process, as discussed in this work. We outline a pathway for transitioning pediatric patients with PCD to adult care.

## 1 Introduction

Over the past decade, there has been a significant increase in the identification and diagnosis of patients with primary ciliary dyskinesia (PCD). The estimated global prevalence, as reported in recent 2022 studies and confirmed in 2025 research, is approximately 1:7500 [1, 2]. With these estimates, 25,000 to 50,000 people in North America are expected to be affected by PCD; however, according to the PCD Registry, only 1000 individuals in North America have received an accurate diagnosis [3]. Bronchiectasis is the third leading cause of lung disease in adults worldwide, and 13% of those cases are thought to be from PCD, though it is still underdiagnosed [4]. More recently, there has been an increased awareness and understanding of PCD in both the pediatric and the adult population, where patients previously diagnosed with idiopathic bronchiectasis are being diagnosed with PCD. This is partly due to the publication of diagnostic guidelines, the recognition of nasal nitric oxide as a screening test, and the increase in the identification of PCD‐causing genes, which has expanded from the first identified PCD‐causing gene (DNAI1) in 1999 [5] to over 55 PCD‐causing genes today [1]. In addition, with the prospect of disease‐specific therapy for certain PCD variants, adult providers may be more inclined to pursue a definitive diagnosis for PCD [6]. Lastly, clinical and research foundations/groups, such as BEAT‐PCD and the PCD Foundation clinical and research network, support clinical and research centers and provide clinical information on PCD worldwide.

The development of transformative genetic modulators for people with CF(pwCF) has led to increasing interest in understanding and managing PCD. There is a paucity of outcome data for pediatric patients with PCD after they reach adulthood and no longer receive treatment at Pediatric Centers. As demonstrated in other chronic illnesses, patients who participate in a PCD transition program are expected to achieve better clinical outcomes and, consequently, better quality of life. Using previously described tools adapted to the unique needs and challenges of patients with PCD, we outline a standardized transition program for implementation across PCDF‐accredited centers. After reviewing several tools, we found the information in a review article by Menon and Lanzkron suitable for adaptation into a process for patients with PC [7, 8].

### 1.1 Methods

This transition pathway was developed following a thorough review of the literature on pediatric chronic diseases that either have a well‐established transition‐to‐care pathway or are in the early stages of one. The six core elements of transition served as the backbone and were adapted to account for PCD‐specific clinical, social, and psychological differences and challenges. To ensure a comprehensive approach, the authors comprise pediatric and adult pulmonology faculty, psychology faculty (all of whom provide direct clinical care to patients with PCD and practice in a PCD Foundation‐accredited center), Med‐Peds faculty, and a Med‐Peds trainee. The parent of a 17‐year‐old patient with PCD reviewed the guidelines and provided feedback, which was incorporated into the final iteration presented in this work.

## 2 Differences Between Pediatric and Adult PCD

### 2.1 Adult and Pediatric PCD Centers in North America

The PCD Foundation website provides information on the Clinical and Research Network, which has increased to 36 accredited Pediatric PCDF clinical centers and 24 Adult PCDF clinical centers, with 15 of these serving as counterparts to the Pediatric PCDF centers [9]. There is no set guideline for the transition from pediatric to adult care for PCD; thus, each center currently follows an individualized transition process. This lack of standardization creates variability and limits the study of outcomes and the optimization of processes for the transition to adult care. Currently, the PCD Foundation is accepting new centers only if they have both pediatric and adult sites to improve transition and access to adult care. Legally, an individual is considered an adult at age 18 in the United States and at 18 or 19 in Canada, depending on the province or territory. For purposes of this transition pathway, adulthood will not be defined by a specific age but rather by a developmental continuum that accounts for cognitive, psychological, and financial circumstances.

### 2.2 Clinical Presentation in Adults With PCD

The prevalence of adult‐diagnosed PCD is unknown. Epidemiologic data on bronchiectasis indicate that 20%–60% of cases are classified as idiopathic; however, it is hypothesized that, in many of these cases, a thorough workup for underlying causes was not completed [10]. In a 2022 study by Shoemark et al., which examined genetic data in patients with severe bronchiectasis, 12% were diagnosed with PCD [4]. Recognizing PCD can be difficult because its presentation may overlap with that of other forms of bronchiectasis, and one of the key diagnostic factors, a history of distress in the newborn period, is unknown in many adult patients. Additionally, accurate diagnostic testing using electron microscopy and genetics can be costly, difficult to access, and carry limited sensitivity. should be. Due to limited diagnostic test sensitivity, supplementary testing such as HSVM, nNO, and IF is necessary but also requires access to specialized centers with significant diagnostic expertise [11]. Clinicians should suspect PCD in adults who have at least two of the following symptoms: unexplained bronchiectasis with chronic rhinosinusitis or ongoing otitis, organ laterality defects, male or female infertility or subfertility, or chronic respiratory symptoms since early childhood [12]. The distribution of bronchiectasis is typically bilateral, affecting the mid and lower lung fields. The prevalence of bacteria such as Pseudomonas aeruginosa and nontuberculous mycobacteria increases with age, potentially requiring more aggressive respiratory therapies. Fertility challenges often present in adulthood when individuals with PCD attempt to have children. Most males are infertile due to sperm immotility (a ciliary structure) or luminal obstruction due to sperm aggregation [13, 14]. Some females with PCD may be infertile or subfertile, though the prevalence is unknown and can be difficult to characterize. Etiologies for female subfertility in PCD are not fully understood, but some hypothesize that limited fallopian tube or endometrial fimbriae motion potentially affects the movement of the egg through the fallopian tubes and uterus [15, 16, 17, 18]. There are reports that ectopic pregnancies may also be more common in females with PCD, but the frequency of this is unclear [19]. Additional data in both males and females with PCD suggest that infertility and/or subfertility correlate with relative gene variant expression in different locations in the body [15].

### 2.3 Mental Health

Despite increasing research to better understand PCD, little is known about the possible mental health co‐morbidities faced by children and adolescents with PCD. Given the similar treatment burden to cystic fibrosis (CF), such as frequent clinic visits with multiple subspecialists, inhaled medications and airway clearance therapy, sinopulmonary exacerbations and hospitalizations, and surgeries, it can be hypothesized that children and adolescents with PCD may experience similar increases in depression and anxiety symptoms as those with CF [20]. A few international studies have examined mental health symptoms in people with PCD (pwPCD). In 2021, a study investigated rates of anxiety and depression in children and adolescents with PCD. The results showed elevated levels of both depression and anxiety symptoms in these groups, as well as their caregivers [21]. These symptoms were associated with poorer health‐related quality of life among pwPCD [21]. A more recent study by Graziano et al. compared mental health outcomes of individuals 12–34 years of age with PCD to those with CF [22]. The study found that people with PCD scored higher on the GAD‐7 and PHQ‐9 assessments, at rates comparable to pwCF [22].

Additionally, people with PCD had higher rates of suicidality compared to those with CF, 9.5% versus 5% [22]. Another Dutch study compared symptoms of depression and anxiety in adolescents with PCD and CF before and after the COVID‐19 pandemic [23]. They found that adolescents with PCD exhibited higher rates of anxiety and depressive symptoms before the pandemic and experienced increased depressive symptoms during it [23]. These findings were similar to those observed in adolescents with CF. Notably, an Italian study conducted during the COVID‐19 pandemic found no significant difference in reported stress among pwPCD parents or care providers compared with controls [24]. The authors concluded that this could be related to the significant decrease in exacerbation rate. While further research is needed to understand the mental health needs of pwPCD, early studies suggest that these individuals experience similar elevations in mental health symptoms as other populations with chronic illnesses, highlighting the importance of incorporating mental health screening and management by mental health professionals into the PCD transition model to adult care.

## 3 The Need for a Transition Process in PCD

A study by Dore et al. examined the experiences of transitioning to adult care among pwPCD aged 18–25 years. This study underscored the need for personalized, integrated support services for people with PCD and for the implementation of healthcare transition frameworks. Additionally, it advocates for repeated assessment of social, educational, and financial support to address changing circumstances during the transition process [25].

Studies of pwCF have shown improved outcomes, including a slower decline in lung function, among patients aged 18 years and older who transition to adult care compared with those who remain under pediatric care [26]. Among more than 10,000 adolescents and young adults, transitions from centers with high participation in the CF RISE program—a structured self‐education and transition program for patients with CF—were associated with better lung function and weight at their first adult clinic visit. Gaps in care exceeding 3 months were associated with reduced lung function [26]. There is limited outcome data for adult patients with PCD who have transitioned from pediatric care.

**Figure 1:**
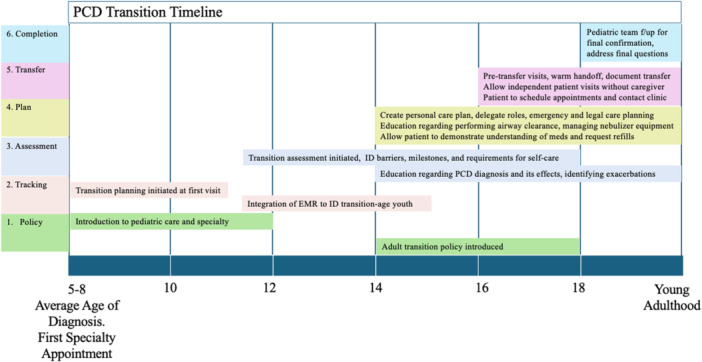
Proposed transition timeline for patients with primary ciliary dyskinesia (PCD).

A pathway to guide PCD centers in transitioning to adult care could enhance readiness across medical, financial, and social domains and establish a framework for research on long‐term outcomes for PCD patients. This would support the development of standardized transition pathways across centers. Figure 1. Illustrates a proposed transition timeline for patients with PCD.

## 4 Barriers to Transition

Barriers to transitioning from pediatric PCD to adult care in the United States include limitations in understanding regarding pathophysiology, therapies, and disease management in general among providers, patients, and caregivers, enabling behaviors among caretakers, and a lack of processes or ability for health care teams to effectively prepare patients and/or caretakers for the transition. Furthermore, challenges with insurance coverage and mental health issues due to chronic disease can also present as barriers to transition.

In addition, there is a paradigm shift between pediatric care models, which tend to be more “concierge‐like,” and adult care models, which require the ability to self‐advocate and direct care. The number of adult pulmonologists specializing in bronchiectasis, especially PCD, is growing but remains relatively limited. Furthermore, pulmonary outpatient clinics typically serve a diagnostically diverse patient population. It is important to recognize that individuals with PCD often require more than pulmonary care; they also need the involvement of otolaryngologists, infectious disease specialists, primary care providers, and, frequently, reproductive specialists. Although nutritional issues impact PCD patients, there is less centralized support compared to patients with CF and pediatric patients with PCD. As lung function declines and respiratory insufficiency increases during adulthood, access to nutritional management represents another area where disparities exist. These circumstances can pose difficulties for pediatric pulmonologists in identifying adult counterparts, thereby ensuring a seamless transition to a team with multidisciplinary expertise in PCD. Establishing the necessary network of healthcare providers can be both challenging and financially burdensome.

### 4.1 Specific Issues With Insurance Coverage in PCD

Insurance coverage from pediatric to adult centers remains a challenge in the United States. Most large pediatric hospitals accept almost all insurance, including state‐sponsored insurance. In contrast, adult hospitals often have narrower insurance networks that do not include state‐sponsored insurance.

For pwPCD, insurance coverage remains an even more complex issue. The diagnostic and treatment guidelines outlined by Shoemark and Shapiro have improved recognition of PCD's phenotype and emphasized the advantages of specific interventions, such as airway clearance [1]. Nevertheless, overall management primarily relies on guidelines derived from other diseases, including cystic fibrosis (CF) and non‐CF bronchiectasis [27]. Currently, there are no United States Food and Drug Administration (FDA)‐approved disease‐modifying medications specifically for PCD, which creates challenges for patients in accessing these medications due to availability, cost, and insurance coverage. Many inhaled antibiotics and mucolytics used in PCD management lack specific indications or supporting studies. Consequently, medication access often involves appeals, which can delay care, increase patients’ disease burden, and lead to burnout among healthcare workers. Patients with CF benefit from comprehensive financial assistance programs supported by a robust research and patient advocacy infrastructure (cff. org), which is not yet accessible to patients with PCD. Furthermore, while patients with CF and other chronic illnesses are automatically eligible for Supplemental Security Income (SSI) or Social Security Disability Insurance (SSDI) in the United States, a diagnosis of PCD, despite being a chronic and disabling condition, does not automatically qualify and necessitates meeting the criteria established by the Social Security Administration (SSA).

A recent study examining the influence of insurance on adolescents' transition to adult healthcare among adolescents with special healthcare needs found that many youths were inadequately prepared to manage insurance matters, including comprehending coverage, networks, copayments, and prior authorizations. Furthermore, gaps in coverage or changes to insurance plans were associated with delays in establishing care and missed appointments [28]. The Journal of Pediatrics recently published research demonstrating that youth with public insurance and those from racial and ethnic minority groups are less likely to have received the recommended transition preparation [29]. Guidelines published for the transition from pediatric to adult care in Sickle Cell disease include key recommendations that could help bridge care gaps, particularly for marginalized populations [30]. Some of the recommendations include assigning a care coordinator or community health worker to help navigate the adult health system and establishing closed‐loop communication between pediatric and adult teams to ensure that patients make their first appointment.

Position statements on healthcare transitions emphasize the importance of incorporating insurance counseling into transition readiness, addressing topics such as understanding coverage and benefits, and ensuring insurance continuity [31]. The Policy Brief on Medicaid & CHIP: Recommendations to Assist Youth and Young Adults with Disabilities Aging Out of Medicaid & CHIP, updated by Got Transition in 2024, recommends that programs develop state Medicaid guidance on outreach and informing Medicaid‐insured youth, their families, and caregivers 12–24 months prior to Medicaid eligibility expiration [2]. Outreach should include informing individuals of the benefit changes that will occur when they no longer qualify for Medicaid as children, along with case management services to connect them with insurance, medical, social, and other support systems in anticipation of aging out. Such guidance should promote coordination among agencies to ensure that accommodations under the Americans with Disabilities Act (ADA) and Section 504 are discussed and implemented prior to aging out, and that continuity of care is maintained following the termination of EPSDT. Moreover, recommendations have been added to ensure that Medicaid members receive educational materials and resources that explicitly communicate the age cutoff, the implications of transitioning out of the child's Medicaid eligibility category, and the availability of alternative Medicaid coverage pathways [2].

## 5 Transition of Care

### 5.1 Roles and Responsibilities

When discussing avenues to help patients with PCD transition from pediatric to adult care, it is essential to first define the roles and responsibilities of everyone involved: the clinical team, the patient, and the caregiver.

1. *The Clinical Team*
  It is the responsibility of the pediatric provider and care team to guide the transition process by regularly evaluating readiness and referring the patient and family to suitable resources to address concerns. Using standardized transition checklists and readiness assessments helps the team identify knowledge gaps [7]. In addition to providing the medical background (the ‘what’) of the disease, it is also important for the provider to help foster a sense of self‐efficacy in navigating the healthcare system (the ‘how’). Referrals to mental health providers, care coordinators, and social workers support addressing contributing factors such as mental health issues, career goals, and stressors. Given the limited number of physicians caring for adult patients with PCD, it is crucial to identify an adult provider and team to work with if the pediatric PCD center is not associated with an adult center.
  Given that the clinical presentation of pediatric patients with Primary Ciliary Dyskinesia (PCD) often involves ear and sinus disease, these patients often follow up with Otolaryngology (ENT) specialists in addition to pulmonologists. A recent study has demonstrated that ear disease remains prevalent among adult patients with PCD [27]. Consequently, involving the ENT physician in the transition process for these patients is imperative.
  The adult healthcare provider and their team also play a significant role, particularly during the later stages of transition, when concerns and apprehensions regarding the initial appointments with the adult healthcare provider may increase. Implementing transition‐specific clinics and designated coordinators, along with maintaining consistent, transparent communication, facilitates a seamless transition to the new clinic and the adult healthcare environment.
  Another suggestion to improve the successful transition to adult care is for combined centers to take the time to develop consistent guidelines across centers. When consistency is not possible, advising patients about differences in care models can help them better advocate for and self‐direct their care. One of the greatest challenges of transition is starting over with a new provider and health system; having both clinical teams acknowledge similarities and differences can help set expectations for patients and families.
2. *The Caregiver*
  Parents or caregivers are responsible for fostering independent behavior in their adolescents, including decision‐making and active involvement throughout the process. A recent study on how parents and caregivers perceive the transition to adulthood for adolescents and young adults with special health care needs found that “my child taking their medications as prescribed” was their top priority. They also preferred to remain actively engaged during their child's shift to adult care [32].
3. *The Patient (disease knowledge, self‐efficacy, adherence, and health literacy)*
*Disease Knowledge*: Patients need a clear understanding of their diagnosis, the benefits of their current therapies, and the importance of adherence. Lack of knowledge about their disease and treatment is a significant barrier to a successful transition.
*Self‐Efficacy and Advocacy*: Refers to the patient's confidence in their ability to manage their condition, communicate with the healthcare team, and navigate the healthcare and insurance systems. In PCD, fragmented care occurs across multiple specialties. Variable insurance coverage and the additional requirement of durable medical equipment companies for airway clearance therapies contribute to challenges with self‐efficacy and advocacy.
*Adherence and Health Literacy*: Patients need to remain adherent to medical treatment despite competing academic, work, and social demands. Due to the burdens of care, as patients pursue normalcy independently, adherence can become more difficult. In addition, the transition to healthcare occurs at a time when many young adults are struggling with the transition to a more independent lifestyle (college, demanding new careers, moving away from home). Health literacy involves the ability to obtain, process, and understand health information to make informed decisions. In PCD, some challenges include the disease's genetic basis and variability, which lead to complex diagnostic language.

### 5.2 The 6 Core Elements of Transition

Got Transition's Six Core Elements of Health Care Transition 3.0 is the widely adopted approach recommended in the 2018 Clinical Report on Health Care Transition from the American Academy of Pediatrics, the American Academy of Family Physicians, and the American College of Physicians [33]. In this case, for patients with PCD, the Six Core Elements may serve as a framework for a structured, patient‐centered transition. The elements are: 1. Transition and Care Policy, 2. Tracking and Monitoring, 3. Transition Readiness, 4. Transition Planning, 5. Transfer of Care, 6. Transfer Completion [34, 35]. Table 1 presents a quick‐reference suggested guide to the 6 core elements of transition for patients with PCD. A checklist adapted from established transition readiness frameworks (CF RISE, TRAQ, and national transition guidelines), designed for longitudinal use with progressive skill acquisition across adolescence and tailored to the multisystem needs of patients with Primary Ciliary Dyskinesia (PCD), is available in the Table S1.

1. *Transition and Care Policy*
  Develop a written transition policy for the PCD clinic that explains the practice's approach to transition and outlines how the care team will support patients and families during this process, assigning specific roles to the youth, the caregiver, and the pediatric and adult healthcare teams.
  a. Describe who will lead the transition process (pediatric pulmonologist, care coordinator, nurse)
  b. Include in the policy a description of how PCD‐specific needs will be addressed (needs for regular chest physiotherapy, ENT and audiology care, fertility counseling, sinus and lung imaging, microbiology, etc.). During this time, it is helpful to map out all the steps for transitioning from pediatric to adult care and to institute a standardized progression for each patient. Okumura and Kleinhenz developed a 10‐step checklist of minimal requirements that a patient and family should be able to meet by the time of transition in the setting of Cystic Fibrosis care [43]. We have modified this checklist to apply to the transition model of patients with PCD.
  c. Share the policy starting between ages 12 and 14 with patients, caregivers, and prospective adult care providers to ensure clear communication and transparency.

**Table 1:** 6 Core elements of transition‐ Quick reference guide.

Transition element	Key actions	PCD‐Specific considerations	Examples of metrics/Outputs	Roles and responsibilities	References
Transition & Care policy	Written transition policy; define roles; identify transition lead; share policy ages 12–14	Airway clearance, ENT/audiology, fertility counseling, imaging, microbiology	Policy document; standardized checklist; early dissemination	Clinical care team	[34]
Tracking & Monitoring	Registry of transition‐aged youth; EMR tracking; milestone monitoring	FEV1, exacerbations, sputum cultures, ENT/audiology, fertility, multispecialty care	Registry completion; time to adult visit; continuity metrics	Clinical care team	[25]
Transition readiness	Assess readiness ages 14–16; evaluate self‐management and advocacy; targeted education	PCD knowledge; airway clearance devices; infection recognition; medication management	TRAQ scores; milestone checklists; education documentation	Clinical care team‐ facilitator	[7, 36, 37, 38, 39, 40, 41]
				Patient‐ Primary participant	
				Caregiver‐ Supportive role	
Transition planning	Individualized plan; goal setting; legal/insurance planning	Adult pulmonology, ENT, fertility care; Medicaid/DME planning; college transitions	Medical summary; emergency plan; insurance counseling	Clinical care team‐ Developer	[7, 42]
				Patient‐ Co‐Developer	
				Caregiver‐ Advisor	
Transfer of care	Direct pediatric–adult communication; transfer summaries; promote independence	Adult providers with PCD or non‐CF bronchiectasis expertise; social supports	Confirmed adult appointment; transfer letter acknowledged	Clinical care teams (Pediatric and Adult)	[7]
				Patient‐ Primary role (leads)	
				Caregiver‐ Supportive role	
Transfer completion	Confirm adult engagement; pediatric follow‐up 3–6 months; collect feedback	Ongoing adult pulmonology, ENT, audiology care	Follow‐up confirmation; satisfaction surveys	Clinical care team Adult (primary role)	[7, 25]
				Clinical Care team Pediatric (safety check)	
				Patient and Caregiver (completers)

2. *Tracking and Monitoring*
  Once the policy is established, tracking and monitoring are essential throughout the process. Identifying transition‐aged youth allows for tracking receipt of the process. For PCD, where disease management is complex and individualized, this step helps maintain continuity and prevent lapses in care [25].
  a. Maintain a registry or list of all patients approaching transition age, systematically tracking their progress and, ideally, integrating these data into the electronic medical record (EMR).
  b. Track key milestones such as when transition conversations were initiated, whether a clinical summary and passport summary have been created, the first adult care referral, and the first adult care visit.
  c. Track PCD‐specific clinical information: lung function (FEV1), frequency of pulmonary exacerbations, adherence, sputum culture results, ENT/audiology status, fertility counseling, and any other specialties involved in care, such as GI.
  d. Use data over time to determine if transition interventions improve continuity and outcomes.
3. *Transition Readiness*
  Assessing readiness is an essential step in the transition process. Narrative reviews of transitioning from chronic illnesses highlight that transition is a process, not a one‐time event, and that early, repeated preparation helps prevent poor outcomes [36].
  The transition team provides education and resources on the necessary skills identified through transition readiness assessments. Few assessments of readiness exist, with the most well‐known being the Transition Readiness Assessment Questionnaire (TRAQ) [38]. By systematically evaluating key areas such as self‐management (e.g., understanding medications, making appointments) and self‐advocacy (e.g., communicating with providers), the TRAQ identifies both strengths and gaps in a patient's readiness for transition [38]. This allows healthcare providers, patients, and families to tailor education and interventions to meet specific needs, rather than using a one‐size‐fits‐all approach.
  Other iterations of transition readiness involve the patient maintaining a continuous checklist of responsibility milestones they should complete (e.g., ‘I can describe how my disability or health condition affects my daily life’) [39]. The fundamental principles of transition readiness have been adapted for other chronic disease populations, such as IBD [40]. The North American Society for Pediatric Gastroenterology, Hepatology, and Nutrition (NASPGHAN) has issued recommendations for providers on evaluating transition readiness based on expert consensus. Their guidelines include seeing adolescent patients without their caregivers to promote independence, providing all relevant medical records to the adult provider to ensure a smoother transfer of medical information, and allowing flexibility in transition timing tailored to the patient's readiness [41]. As outlined by Menon and Afzali, many other efforts aim to facilitate the pediatric‐to‐adult transition, including the American College of Physicians' “Pediatric to Adult Care Transitions Initiative,” which is developing disease‐specific transition toolkits. However, a specific one for PCD does not yet exist [7].
  a. Starting between the ages of 14 and 16, consider the patient's developmental stage and begin assessing the patient's knowledge of PCD and self‐management skills. For example, evaluate their understanding of their diagnosis and the long‐term disease implications, such as progression of oto‐sino‐pulmonary disease and fertility.
  b. Assess psychosocial readiness, including awareness of differences in adult care.
  c. Evaluate the patient's competence in medication management, including the rationale of therapies, airway clearance routine, including different options for chest physiotherapy (high frequency chest wall oscillation, oscillating Positive Expiratory Pressure (PEP) devices, etc.), handling symptoms, scheduling appointments, calling the provider's office for guidance, symptom management, questions, and refill requests. Provide ongoing targeted education (airway clearance, use of nebulizers, understanding microbiology results and importance, immunizations, sinus care.
    and how to discuss with youth and caregivers their self‐care needs, and how to utilize healthcare services.
  d. Identify and address potential barriers to transition, recognize disease‐related knowledge gaps (such as developmental and psychosocial obstacles), and review the complexities of an individual's disease burden. For PCD patients, this might include learning airway clearance techniques, recognizing signs of infection, and understanding the importance of routine monitoring. [7, 25],

4. *Transition Planning*
  Transition planning involves developing a comprehensive, personalized plan that includes the youth's goals and prioritized actions, as well as legal changes in decision‐making, privacy, consent, self‐advocacy, and access to information. Proper goal setting, outlining specific actions, delegating responsibilities between youth and caregiver, and setting a timeline for each goal are essential components of this stage. Planning with youths and caregivers to optimize the timing of transfer is also ongoing. It is also an opportunity to transfer medical summaries, emergency care instructions, and discussions about legal and insurance issues. For PCD, this plan should address the need for ongoing respiratory, ENT, and fertility care, and identify adult providers with expertise in PCD or related disorders. Menon and Afzali also comment in their work that they use a patient navigator to help organize the adult transition, and other studies have reported the utility of having a designated transition coordinator. [7, 42],
  a. Create a PCD‐specific medical care summary and an emergency care plan to accompany the patient during transfer to adult care. It should include diagnosis details when available, genetic variants, ciliary transmission electron microscopy (TEM) results, high speed video microscopy results, lung function trends, history of pulmonary exacerbations/hospitalizations, airway clearance regimen (frequency, type of physiotherapy, devices), immunization history, respiratory cultures (including organisms and sensitivities), ENT disease history, audiology results, fertility counseling, current medications and doses, allergy and surgical history. Diagnostic confirmation makes it easier for adult clinicians and new plans to justify ongoing coverage.
  b. Include complications, side effects of any therapies, or less common associated co‐morbidities, such as bleeding with certain mucolytics, a history of pneumothorax, cholesteatoma, or hydrocephalus.
  c. Start insurance literacy and benefits counseling early. For example, what is Medicaid versus CHIP versus commercial, how to find in‐network adult pulmonologists and ENTs, how prior authorization works for airway clearance devices, nebulized medications, and surgeries.
  d. Flag PCD patients who are on Medicaid by ages 17‐18 and work with social work and benefits staff to map out when EPSDT and child eligibility end in your state. Identify adult eligibility pathways (SSI, waiver expansion) applicable to that family. Initiate redetermination applications before aging out to avoid a coverage gap.
  e. For patients going to college out of state or moving, check the new state's adult Medicaid coverage for respiratory durable medical equipment (DME), ENT, hearing, and preferred drug lists, anticipate which therapies will need a new prior authorization or may not be covered, and when possible, plan for a bridge to include extra supplies.
  f. Define a transition plan of care with goals and guidelines. For example, by age 17, schedule the patient's first adult pulmonology visit; by age 18, establish ENT/audiology care; and by age 20, provide fertility counseling.
5. *Transfer of Care*
  The actual transfer of care is carefully coordinated. Pediatric and adult providers communicate directly, sharing detailed medical histories and treatment plans. The patient and family are supported in establishing new relationships with adult providers and in arranging pre‐transfer visits to ensure a smooth handoff rather than an abrupt break.
  a. Ensure that the adult care team has access to clinicians experienced with PCD. When an adult PCD‐accredited center is not available, identify adult pulmonologists with experience in non‐CF bronchiectasis and/or rare lung diseases, as well as adult ENT, audiology, and fertility providers. PCD is a rare disease, so this may require referral to a specialized center rather than general adult pulmonary practices
  b. In addition to the medical summary, transfer patient and caregiver contact information, proffered airway clearance regimen, social challenges in care such as transportation, support system, etc.
  c. Confirm the date for the first adult clinician appointment with the transition team. Best practices recommend preparing a letter with the transfer package, sending it to the adult clinician, and confirming receipt. It is also a good opportunity to let the patient schedule their own appointment and to hold some initial appointments without the caregiver, if possible. Ideally, the patient should be able to pick up their own medications from the pharmacy and request refills.
6. *Transfer Completion*
  Finally, after the first visit with the adult provider, verify that the transfer is complete, ensuring that follow‐up has been established with the adult pulmonologist, ENT, and other required specialists.
  a. The pediatric team follows up within 3 to 6 months to confirm that the young adult has successfully engaged with their new care team and to address any lingering questions or barriers. It is also helpful for the pediatric team to hold multidisciplinary meetings to assess progress and to discuss medical changes with the patient and family.
  b. Feedback from patients and families is used to improve the process for future transitions. To do this, a survey can be sent to both the patient and their family to assess how well the transition went. Some areas to consider include the helpfulness of medical surveys, communication gaps, gaps in adult care, and any other additional support.

For PCD patients, applying these six elements helps address the unique challenges of living with a rare, multisystem disease. It supports identity development, encourages independence, and creates a dependable support network as they transition into adulthood—factors shown to be essential for a successful transition and continued health.

An ideal longitudinal transition process begins in early childhood with introduction to specialty care and early transition planning, incorporating progressively increasing responsibility across developmental stages. Although many patients experience delayed diagnosis and may establish care with a PCD provider at older ages, this framework remains adaptable and can be flexibly applied to meet each patient's developmental level and individual needs.

## 6 Future Directions

To the best of our knowledge, this is one of two articles to explore barriers and limitations to pediatric PCD transition care and the first to propose a transition model for pediatric PCD patients transitioning to adult care. The appropriate transition model for this population should be established through future research that examines the outcomes of this process and ensures that input from pediatric and adult patients and their families is considered when implementing generalized transition models. Given the limited number of adult centers, transition processes will need to account for community and local resources. Pediatric providers caring for this population will need to play an active role in educating and engaging the adult health care team to develop these transition models in ways that are sustainable, acceptable to the health care team, and, most importantly, to the patient population.

## Author Contributions

**Abel De Castro:** investigation, writing – original draft, writing – review and editing, formal analysis, data curation. **Melanie Sue Collins:** writing – review and editing, data curation, formal analysis. **Raksha Jain:** conceptualization, writing – review and editing, data curation, formal analysis, investigation. **Jennifer B. Walsh:** writing – review and editing. **Juliana Alba‐Suárez:** writing – review and editing, data curation, formal analysis, investigation. **Yadira M. Rivera‐Sánchez:** conceptualization, investigation, writing – review and editing, writing – original draft, formal analysis, supervision, data curation, project administration.

## Funding

The authors have nothing to report.

## Conflicts of Interest

The authors declare no conflicts of interest.

## Supporting information


Supporting File 1

Supporting File 2

## Data Availability

The data that support the findings of this study are available from the corresponding author upon reasonable request.
