# Supplementary material for: Primary Ciliary Dyskinesia: A Suggested Pathway to Transition From Pediatric to Adult Care
Source: Pediatr Pulmonol. 2026 Aug 13;61(8):e71780. doi: 10.1002/ppul.71780 (PMC13470829; doi:10.1002/ppul.71780)
Supplement: Supplementary file 1 [file PPUL-61-0-s001.docx]

**Supplementary material: The Six Core Elements of Transition adaptation for patients with PCD: Expanded Version.**

1. *Transition and Care Policy*

Develop a written transition policy for the PCD clinic that explains the practice’s approach to transition and outlines how the care team will support patients and families during this process, assigning specific roles to the youth, the caregiver, and the pediatric and adult healthcare teams.

a. Describe who will lead the transition process (pediatric pulmonologist, care coordinator, nurse)

b. Include in the policy a description of how PCD-specific needs will be addressed (needs for regular chest physiotherapy, ENT and audiology care, fertility counseling, sinus and lung imaging, microbiology, etc.). During this time, it is helpful to map out all the steps for transitioning from pediatric to adult care and to institute a standardized progression for each patient. Okumura and Kleinhenz developed a 10-step checklist of minimal requirements that a patient and family should be able to meet by the time of transition in the setting of Cystic Fibrosis care^s1^. We have modified this checklist to apply to the transition model of patients with PCD.

c. Share the policy starting between ages 12-14 with patients, caregivers, and prospective adult care providers to ensure clear communication and transparency.

2. *Tracking and Monitoring*

Once the policy is established, tracking and monitoring are essential throughout the process. Identifying transition-aged youth allows for tracking receipt of the process. For PCD, where disease management is complex and individualized, this step helps maintain continuity and prevent lapses in care.^s2^

a. Maintain a registry or list of all patients approaching transition age, systematically tracking their progress and, ideally, integrating it into the electronic medical records (EMR).
b. Track key milestones such as when transition conversations were initiated, whether a clinical summary and passport summary have been created, the first adult care referral, and the first adult care visit.
c. Track PCD-specific clinical information- lung function (FEV1), frequency of pulmonary exacerbations, adherence, sputum culture results, ENT/audiology status, fertility counseling, and any other specialties involved in care, such as GI.
d. Use data over time to determine if transition interventions improve continuity and outcomes.

3. *Transition Readiness*

Assessing readiness is an essential step in the transition process. Narrative reviews of transitioning from chronic illnesses highlight that transition is a process, not a one-time event, and that early, repeated preparation helps prevent poor outcomes^s3^.

The transition team provides education and resources on the necessary skills identified through transition readiness assessments. Few assessments of readiness exist, with the most well-known being the Transition Readiness Assessment Questionnaire (TRAQ)^s4^. By systematically evaluating key areas such as self-management (e.g., understanding medications, making appointments) and self-advocacy (e.g., communicating with providers), the TRAQ identifies both strengths and gaps in a patient’s readiness for transition^s5^. This allows healthcare providers, patients, and families to tailor education and interventions to meet specific needs, rather than using a one-size-fits-all approach.

Other iterations of transition readiness involve the patient maintaining a continuous checklist of responsibility milestones they should complete (e.g., “I can describe how my disability or health condition affects my daily life”)^s6^. The fundamental principles of transition readiness have been adapted for other chronic disease populations, such as IBD^s7^. The North American Society for Pediatric Gastroenterology, Hepatology, and Nutrition (NASPGHAN) has issued recommendations for providers on evaluating transition readiness based on expert consensus. Their guidelines include seeing adolescent patients without their caregivers to promote independence, providing all relevant medical records to the adult provider to ensure a smoother transfer of medical information, and allowing flexibility in transition timing tailored to the patient's readiness ^s8^. As outlined by Menon and Anfzali, many other efforts aim to facilitate the pediatric-to-adult transition, including the American College of Physicians' “Pediatric to Adult Care Transitions Initiative,” which is developing disease-specific transition toolkits. However, a specific one for PCD does not yet exist ^s9^.

a. Starting in early adolescence, between the ages of 14 and 16, considering the patient’s developmental stage, begin assessing the patient’s knowledge of PCD and self-management skills. For example, evaluate their understanding of their diagnosis and the long-term disease implications, such as progression of oto-sino-pulmonary disease and fertility.
b. Assess psychosocial readiness, including awareness of differences in adult care.
c. Evaluate the patient’s competence in medication management, including the rationale of therapies, airway clearance routine, including different options for chest physiotherapy (high frequency chest wall oscillation, oscillating Positive Expiratory Pressure (PEP) devices, etc.), handling symptoms, scheduling appointments, calling the provider’s office for guidance, symptom management, questions, and refill requests. Provide ongoing targeted education (airway clearance, use of nebulizers, understanding microbiology results and importance, immunizations, sinus care.

and how to discuss with youth and caregivers their self-care needs, and how to utilize healthcare services.

d. Identify and address potential barriers to transition, recognize disease-related knowledge gaps (such as developmental and psychosocial obstacles), and review the complexities of an individual’s disease burden. For PCD patients, this might include learning airway clearance techniques, recognizing signs of infection, and understanding the importance of routine monitoring. ^s2,9^

4. *Transition Planning*

Transition planning involves developing a comprehensive, personalized plan that includes the youth’s goals and prioritized actions, as well as legal changes in decision-making, privacy, consent, self-advocacy, and access to information. Proper goal setting, outlining specific actions, delegating responsibilities between youth and caregiver, and setting a timeline for each goal are essential components of this stage. Planning with youths and caregivers to optimize the timing of transfer is also ongoing. It is also an opportunity to transfer medical summaries, emergency care instructions, and discussions about legal and insurance issues. For PCD, this plan should address the need for ongoing respiratory, ENT, and fertility care, and identify adult providers with expertise in PCD or related disorders. Menon and Anfzali also comment in their work that they use a patient navigator to help organize the adult transition, and other studies have reported the utility of having a designated transition coordinator. ^s9,10^

a. Create a PCD-specific medical care summary and an emergency care plan to accompany the patient during transfer to adult care. It should include diagnosis details (genetic variants, ciliary transmission electron microscopy (TEM) results, lung function trends, history of pulmonary exacerbations/hospitalizations, airway clearance regimen (frequency, type of physiotherapy, devices), immunization history, respiratory cultures (including organisms and sensitivities), ENT disease history, audiology results, fertility counseling, current medications and doses, allergy and surgical history. Diagnostic confirmation makes it easier for adult clinicians and new plans to justify ongoing coverage.
b. Include complications, side effects of any therapies, or less common associated co-morbidities, such as bleeding with certain mucolytics, a history of pneumothorax, cholesteatoma, or hydrocephalus.
c. Start insurance literacy and benefits counseling early. For example, what is Medicaid vs. CHIP vs. commercial, how to find in-network adult pulmonologists and ENTs, how prior authorization works for airway clearance devices, nebulized medications, and surgeries.
d. Flag PCD patients who are on Medicaid by ages 17-18 and work with social work and benefits staff to map out when EPSDT and child eligibility end in your state. Identify adult eligibility pathways (SSI, waiver expansion) applicable to that family. Initiate redetermination applications before aging out to avoid a coverage gap.
e. For patients going to college out of state or moving, check the new state’s adult Medicaid coverage for respiratory durable medical equipment (DME), ENT, hearing, and preferred drug lists, anticipate which therapies will need a new prior authorization or may not be covered, and when possible, plan for a bridge to include extra supplies
f. Define a transition plan of care with goals and guidelines. For example, by age 17, schedule the patient’s first adult pulmonology visit; by age 18, establish ENT/audiology care; and by age 20, provide fertility counseling.

5. *Transfer of Care*

The actual transfer of care is carefully coordinated. Pediatric and adult providers communicate directly, sharing detailed medical histories and treatment plans. The patient and family are supported in establishing new relationships with adult providers and in arranging pre-transfer visits to ensure a smooth handoff rather than an abrupt break.

a. Ensure that the adult care team has access to clinicians experienced with PCD. When an adult PCD-accredited center is not available, identify adult pulmonologists with experience in non-CF bronchiectasis and/or rare lung diseases, as well as adult ENT, audiology, and fertility providers. PCD is a rare disease, so this may require referral to a specialized center rather than general adult pulmonary practices
b. In addition to the medical summary, transfer patient and caregiver contact information, proffered airway clearance regimen, social challenges in care such as transportation, support system, etc.
c. Confirm the date for the first adult clinician appointment with the transition team. Best practices recommend preparing a letter with the transfer package, sending it to the adult clinician, and confirming receipt. It is also a good opportunity to let the patient schedule their own appointment and to hold some initial appointments without the caregiver, if possible. Ideally, the patient should be able to pick up their own medications from the pharmacy and request refills.

6. *Transfer Completion*

Finally, after the first visit with the adult provider, verify that the transfer is complete, ensuring that follow-up has been established with the adult pulmonologist, ENT, and other required specialists.

a. The pediatric team follows up within 3 to 6 months to confirm that the young adult has successfully engaged with their new care team and to address any lingering questions or barriers. It is also helpful for the pediatric team to hold multidisciplinary meetings to assess progress and to discuss medical changes with the patient and family.
b. Feedback from patients and families is used to improve the process for future transitions. To do this, a survey can be sent to both the patient and their family to assess how well the transition went. Some areas to consider include the helpfulness of medical surveys, communication gaps, gaps in adult care, and any other additional support.

For PCD patients, applying these six elements helps address the unique challenges of living with a rare, multisystem disease. It supports identity development, encourages independence, and creates a dependable support network as they transition into adulthood—factors shown to be essential for a successful transition and continued health.

S1.	Okumura MJ, Kleinhenz ME. Cystic Fibrosis Transitions of Care: Lessons Learned and Future Directions for Cystic Fibrosis. *Clin Chest Med.* 2016;37(1):119-126.

S2.	Dore R, Nizza IE, Mitchison HM, Lewis C. 'Don't let it hold you back' - The experience of transition to adulthood in young people with primary ciliary dyskinesia: An interpretative phenomenological analysis. *J Health Psychol.* 2024;29(9):1029-1045.

S3.	Willis LD. Transition From Pediatric to Adult Care for Young Adults With Chronic Respiratory Disease. *Respiratory Care.* 2020;65(12):1916-1922.

S4.	Wood DL, Sawicki GS, Miller MD, et al. The Transition Readiness Assessment Questionnaire (TRAQ): its factor structure, reliability, and validity. *Acad Pediatr.* 2014;14(4):415-422.

S5.	Sawicki GS, Lukens-Bull K, Yin X, et al. Measuring the transition readiness of youth with special healthcare needs: validation of the TRAQ--Transition Readiness Assessment Questionnaire. *J Pediatr Psychol.* 2011;36(2):160-171.

S6.	Gibson RW RJ, Raum ND. Envisioning My Future. A Young Person’s Guide to Healthcare Transition., ICHP at the University of Florida.

S7.	Huang JS, Tobin A, Tompane T. Clinicians poorly assess health literacy-related readiness for transition to adult care in adolescents with inflammatory bowel disease. *Clin Gastroenterol Hepatol.* 2012;10(6):626-632.

S8.	Baldassano R, Ferry G, Griffiths A, Mack D, Markowitz J, Winter H. Transition of the patient with inflammatory bowel disease from pediatric to adult care: recommendations of the North American Society for Pediatric Gastroenterology, Hepatology and Nutrition. *J Pediatr Gastroenterol Nutr.* 2002;34(3):245-248.

S9.	Menon T, Afzali A. Inflammatory Bowel Disease: A Practical Path to Transitioning From Pediatric to Adult Care. *Am J Gastroenterol.* 2019;114(9):1432-1440.

S10.	Annunziato RA, Baisley MC, Arrato N, et al. Strangers headed to a strange land? A pilot study of using a transition coordinator to improve transfer from pediatric to adult services. *J Pediatr.* 2013;163(6):1628-1633.
