# Supplementary material for: Primary Ciliary Dyskinesia: A Suggested Pathway to Transition From Pediatric to Adult Care
Source: Pediatr Pulmonol. 2026 Aug 13;61(8):e71780. doi: 10.1002/ppul.71780 (PMC13470829; doi:10.1002/ppul.71780)
Supplement: Supplementary file 2 [file PPUL-61-0-s002.docx]

**Supplementary material**

**Table S1:** Adapted from established transition readiness frameworks (CF RISE, TRAQ, and national transition guidelines) and tailored to the multisystem needs of patients with Primary Ciliary Dyskinesia (PCD). It is designed for longitudinal use with progressive skill acquisition across adolescence.

| **Domain** | **Skill / Responsibility** | **14–15 yrs Introduced** | **16–17 yrs Practicing** | **18+ yrs Independent** | **Notes / Support Needed** |
| --- | --- | --- | --- | --- | --- |
| **Responsibility** | Can explain PCD diagnosis and how it affects lungs, sinuses, ears, and fertility | ☐ | ☐ | ☐ | Primary pulmonologist, primary pediatrician, primary adult physician, ENT |
| **Responsibility** | Knows diagnostic history (genetics, TEM, nasal NO if available) | ☐ | ☐ | ☐ | Primary pulmonologist, primary pediatrician, ENT |
| **Responsibility** | Recognizes symptoms of pulmonary and sinus exacerbations | ☐ | ☐ | ☐ | Primary pulmonologist, primary pediatrician, respiratory therapist, ENT |
| **Independence** | Performs airway clearance independently (HFCWO, PEP, manual CPT) | ☐ | ☐ | ☐ | Primary pulmonologist, respiratory therapist |
| **Independence** | Prepares, cleans, and troubleshoots nebulizer equipment | ☐ | ☐ | ☐ | Primary pulmonologist, respiratory therapist |
| **Independence** | Participates in clinic visits without caregiver present | ☐ | ☐ | ☐ | Primary pulmonologist, primary pediatrician, primary adult physician |
| **Self-Care** | Knows medication names, doses, and purpose | ☐ | ☐ | ☐ | Primary pulmonologist, primary adult physician |
| **Self-Care** | Understands sputum cultures and microbiology results | ☐ | ☐ | ☐ | Primary pulmonologist, primary pediatrician, primary adult physician |
| **Self-Care** | Requests medication refills and manages pharmacy communication | ☐ | ☐ | ☐ | Primary pulmonologist, primary adult physician |
| **Education & Advocacy** | Schedules appointments and pulmonary testing | ☐ | ☐ | ☐ | Primary pulmonologist, primary adult physician |
| **Education & Advocacy** | Contacts clinic for worsening symptoms or questions | ☐ | ☐ | ☐ | Primary pulmonologist, primary pediatrician, primary adult physician |
| **Education & Advocacy** | Understands differences between pediatric and adult care models | ☐ | ☐ | ☐ | Primary pulmonologist, primary pediatrician, primary adult physician |
| **Education & Advocacy** | Understands insurance type and how coverage may change after age 18 | ☐ | ☐ | ☐ | Primary pulmonologist, primary adult physician |

**Suggested Use**

• Review annually beginning at ages 14–15
• Use to guide targeted education and transition planning
• Expect progression across age bands rather than completion at a single visit
• Reassess prior to transfer to adult care
